# The effect of considering eye movement time in evaluating the efficiency of attentional networks

**DOI:** 10.1002/pchj.734

**Published:** 2024-02-01

**Authors:** Hossein Akbari‐Lalimi, Seyed Ali Shafiei, Mahdi Momennezhad, Hoda Zare, Ali Talaei, Shahrokh Naseri

**Affiliations:** ^1^ Department of Medical Physics, Faculty of Medicine Mashhad University of Medical Sciences Mashhad Iran; ^2^ Neuroscience Research Center Qom University of Medical Sciences Qom Iran; ^3^ Nuclear Medicine Research Center Mashhad University of Medical Sciences Mashhad Iran; ^4^ Medical Physics Research Center Mashhad University of Medical Sciences Mashhad Iran; ^5^ Psychiatry and Behavioral Sciences Research Center Mashhad University of Medical Sciences Mashhad Iran

**Keywords:** alerting, attention, executive, eye movement, orienting

## Abstract

The attention network test (ANT) is a tool for assessing the executive, alerting, and orienting components of attention. However, conflicting findings exist regarding the nature and correlation between attention networks. This study aims to investigate the influence of eye movement time on the assessment of attention network efficiency. Forty male students, with an average age of 20.8 ± 1.3 years, participated in the study. The revised attention network test was conducted concurrently with the recording of the electrooculogram signal. The electrooculogram signal was used to estimate eye placement time on target stimuli. Considering eye movement time for calculating the score of each network was proposed as a novel method. The study explored the nature of attention networks and their relationships, and revealed significant effects for attention networks with and without considering the eye movement time. Additionally, a significant correlation is observed between the alerting and orienting networks. However, no significant correlation is found between attention networks using the proposed method. Considering eye movement time alters the assessment of attention network efficiency and modifies the correlation among attention networks.

## INTRODUCTION

Posner's attention theory proposes that attention can be divided into three distinct networks: alerting, orienting, and executive control (Fan et al., 2002; 2005; 2009; Fan & Posner, 2004; Petersen & Posner, 2012; Posner & Petersen, 1990; Posner & Rothbart, 2007). The alerting network is responsible for acquiring and maintaining a state of high sensitivity to stimuli. The orienting network involves selecting relevant information from sensory inputs. The executive control network encompasses mechanisms for monitoring and resolving cognitive, sensory, and response difficulties (Fan et al., 2002).

Studies have found that these three attention networks are associated with distinct anatomical areas (Fan et al., 2005). In 2001, the attention network test (ANT) was developed as a single‐session evaluation of the efficiency of the alerting, orienting, and executive control networks (Fan et al., 2001; Fossella, Posner, et al., 2002). The test was fully introduced and evaluated in 2002 (Fan et al., 2002). The ANT is a combination of the spatial cue test (Posner, 1980) and the flanker flash test (Eriksen & Eriksen, 1974).

Since its introducing, researchers have made modifications to the original ANT to suit their specific research objectives, resulting in different versions of the test (Callejas et al., 2004; Fan et al., 2009; Roberts et al., 2006; Rueda et al., 2004). Consequently, the task and its modified versions have been widely used in various studies (de Souza Almeida et al., 2021).

One of the topics under discussion is the interdependence of attention networks. If there is a significant correlation between the scores of attention networks in study samples, it indicates that these networks are dependent. Conversely, an insignificant correlation indicates their independence. The original report of ANT did not find a significant relationship between attention networks (Fan et al., 2002). Other studies have also reported the independence of attention networks (Fan et al., 2009; Gooding et al., 2006; Huang et al., 2021; Ishigami & Klein, 2010; Jin & Lin, 2022; LaCroix et al., 2021; Medina & Barraza, 2019; Noh et al., 2012; Sørensen et al., 2019; Wang et al., 2005; Wu et al., 2021).

In addition to their independence, there are three distinct anatomical networks associated with attention components (Fan et al., 2005). Each attention network is also related to a separate set of neural oscillations (Fan et al., 2007). However, some studies have reported significant relationships between certain networks, and not all studies confirm the independence of attention networks. For example, significant correlations have been observed between the alerting and orienting networks in both healthy and diseased individuals (Kalia et al., 2018; Lehtonen, 2007; Medina & Barraza, 2019; Ren et al., 2021; Vig, 2018; Wu et al., 2021). Also, Macleod et al. analyzed data from 1141 samples across 15 different studies and found a significant relationship between these networks (MacLeod et al., 2010). Similarly, significant correlations have been observed between the alerting and executive control networks in various studies (Fossella, Sommer, et al., 2002; Jeong, 2021; Kalia et al., 2018; Ren et al., 2021; Wu et al., 2021). Some studies have also found significant correlations between the orienting and executive networks (LaCroix et al., 2021; Wu et al., 2020; Zhang et al., 2020).

Drawing conclusions about the dependence or independence of attention networks is challenging due to the divergent findings in different studies. While there is ample evidence supporting the independence of attention networks, some studies have reported their dependence to each other. Furthermore, the interaction between these networks has been investigated and documented in various studies (Barclay et al., 2020; Fan et al., 2002; 2009; Fuentes & Campoy, 2008; Mackie et al., 2013; McConnell & Shore, 2011; Sørensen et al., 2018).

Based on the study conducted by Kulke et al. (2016), similarities were observed in the neural mechanisms involved in covert and overt attention shift tasks. The discrepancies between these two forms of attention are commonly attributed to the saccade inhibition effect. Therefore, it can be concluded that when investigating covert attention, the inhibitory effect, in addition to the orienting effect, plays a significant role. Consequently, this study aimed to investigate overt attention.

In the studies, the reaction time difference between specific cue conditions or congruency conditions was used to calculate the scores of attention networks. It is assumed that the effects of cue conditions or congruency conditions not involved in the calculation of a particular network score are removed through randomization. While this assumption is generally valid, randomization does not always guarantee the elimination of unwanted effects. The ANT assumes that during each trial's time intervals, the functions associated with the three attention networks are in operation. However, it is acknowledged that there may be additional factors unrelated to these three networks. For example, factors such as eye movement time and the interval between choosing the direction of the central arrow and pressing the key can be considered. This study aims to purify the scores of each network by taking into account the eye movement time. Furthermore, the study investigates the relationship between attentional networks, both with and without considering eye movement time.

## MATERIALS AND METHODS

### Participants

A total of 40 young individuals, with an average age of 20.8 years (*SD* = 1.3, range = 19–25, median = 21), were included in this research. All participants were right‐handed males (self‐reported) and were selected from volunteers who were university students. It was ensured that the participants were not under the influence of drugs or alcohol during the experiment. Informed consent forms were obtained from all participants. The present study received approval from the Ethics Committee of Mashhad University of Medical Sciences.

### Electrooculogram data recording

The electrooculogram (EOG) signal was recorded using three separate electrodes. Two electrodes were placed in the outer corner of the left and right eyes, while one electrode was positioned at the bottom of the right eye. These electrodes were connected to the g.HIamp or g.USBamp amplifiers from g.tec (Austria). The maximum resistance of the active electrodes was set at 50 kΩ, and the sampling frequency was 512 Hz. A 50 Hz notch filter was applied to suppress line noise.

To ensure accurate timing and record different stimuli in the task, as well as the subject's responses, a parallel port and g.TRIGbox system from g.tec (Austria) were used. These systems recorded the stimuli appearance time and type, as well as the time and type of the subject's response, in synchronization with the EOG signal.

### Revised attention network test (ANT‐R)

The ANT‐R was conducted in a quiet, dimly lit room at the National Brain Mapping Laboratory. The EOG signals were recorded simultaneously with the task, following the method described in the previous section. The times of appearance of targets and cues, as well as the subject's responses, were sent to the signal recording system and recorded in synchronization with the electrical signals.

To present the task stimuli and capture the subjects' responses, we utilized the Psychtoolbox, which is a MATLAB toolbox (Kleiner et al., 2007). A 19‐inch monitor with a nominal frequency of 60 Hz was used for the task presentation. The distance between the subject's eyes and the screen was set at 60 cm. To facilitate ease of response, the keyboard was employed, with clearly labeled keys specifically designated for the experiment.

Prior to commencing the task, the subjects were instructed to carefully read the task instructions, and any queries or uncertainties they had were addressed. For this study, we employed the ANT‐R, an assessment originally introduced by Fan et al. (2009). The study incorporated various conditions, including no cue, double cue, and single cue, as well as congruent and incongruent target conditions.

The revised attention network test, ANT‐R, thoughtfully preserves the core attention‐related networks and mechanisms of the original test, while integrating additional elements for a more comprehensive evaluation. Notably, it introduces the variable cue‐target interval, effectively neutralizing any potential influence stemming from the predictability of the precise timing for target stimulus presentation.

**FIGURE 1:**
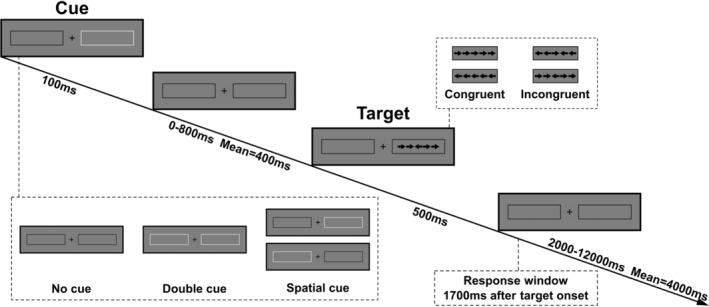
Schematic of the revised attention network test. After a variable duration, the target arrow and four flanker arrows on the left and right sides (congruent or incongruent) are presented for 500 ms. Participants are required to respond to the direction of the target arrow. The duration of post‐target fixation varies between 2000 and 12,000 ms.

The specific details of the ANT‐R task employed in this study are illustrated in Figure 1. During the task, a fixation cross was positioned at the center of the screen against a gray background, with two rectangular boxes flanking it on both sides. In each trial, if a cue was present, one or both of the boxes would briefly flash for 100 ms. In the absence of a cue, the boxes remained unchanged. Following a variable interval, with an average duration of 400 ms (ranging from 0, 400, to 800 ms), the target stimuli appeared and were displayed for 500 ms.

The target stimuli consisted of five arrows arranged side by side, each pointing either to the right or left. In each trial, these five arrows appeared within either the right or left box. The objective of the task was to identify and select the direction of the central arrow. In the congruent condition, the direction of the four non‐central arrows matched that of the central arrow. In the incongruent condition, the direction of the four non‐central arrows was opposite to that of the central arrow.

Each arrow had dimensions of 1.34° in length and 0.75° in width, while the distance between the arrows measured 0.08°. Thus, the total visual angle covered by the five arrows amounted to 7.02°. The distance between the center of the target arrow and the center of the image or cross was 5.05°.

The time interval between the presentation of stimuli and the start of the subsequent trial varied. This interval ranged from 2000 to 12000 ms, with specific values within this range including 2000, 2250, 2500, 2750, 3000, 3250, 3500, 3750, 4000, 4250, 4750, and 12000 ms. The average duration of this interval was 4000 ms. Each trial had an average duration of 5000 ms, and the response time window for the subjects was set at 1700 ms.

The test comprised four runs, with each run consisting of 60 trials. The total duration of the four runs amounted to 20 min. Between runs, the subjects were allowed to rest for as long as they desired. Out of the total 240 trials, 120 trials featured congruent flanker stimuli, while the remaining 120 trials featured incongruent flanker stimuli.

Furthermore, out of the total 240 trials, 40 trials were presented without any cues, 40 trials had only double cues (both boxes flashing), and 160 trials featured single cues (one box flashing). Within the 160 trials with single cues, 120 trials included valid spatial cues, while 40 trials included invalid spatial cues. According to the study by Fan et al. (2009), the probability of the valid spatial cue is equal to the combined probability of the invalid spatial cue, double cue, and no cue conditions. The double cue provides temporal information regarding the appearance of the target stimuli, while the valid spatial cue provides both temporal and spatial information about the appearance of the target stimuli. Throughout the revised attention network test, the EOG signals were simultaneously recorded.

During the task instructions, participants were instructed to identify and select the direction of the central arrow using the designated predetermined keys once the arrows became visible. Clear explanations were provided regarding the flashing conditions of the boxes and the corresponding meaning of each condition. Participants were instructed to respond both quickly and accurately. Notably, in this test, participants were allowed to freely move their eyes.

### Processing and calculation of cognitive functions

To analyze the cognitive functions using the ANT‐R, the recorded data from the parallel port were utilized. The performance of the alerting, orienting, and executive networks, which represent the three functional components of the attention process, was calculated. To account for practice effects, the first 40 trials of each subject were excluded from the analysis.

The scores for the attention networks were calculated using two different methods: the default method and the method based on eye movement. The default method evaluated the attention network scores by comparing reaction times in specific target or cue conditions with those in other conditions. Specifically, the alerting network score was calculated as the difference in reaction time between the no cue condition and the double cue condition. The orienting network score was determined by the difference in reaction time between the double cue condition and the valid spatial cue condition. The executive network score was obtained by calculating the difference in reaction time between the congruent condition and the incongruent condition. Additionally, for each network, the difference in response accuracy between the aforementioned conditions was also calculated.

Alerting=RTnocue–RTdoublecue.

Orienting=RTdoublecue–RTvalid spatialcue.

Executive=RTincongruent–RTcongruent.

$\mathrm{RT}=\text{the time interval starting from the appearance of the target stimuli to the}\;{\text{subject}}^{'}s\;\text{response}.$

In the process of calculating the scores for the attention networks using the eye movement‐based approach (proposed method), we utilized the eye placement moment on the target, which had been previously extracted. For the executive network, we considered the difference in modified reaction time between the congruent and incongruent conditions. In the proposed method, the modified reaction time was defined as the time interval between the moment the subject's eyes landed on the stimulus and their subsequent response.

The alerting network score was determined by measuring the difference in time interval between the display of the target stimulus and the placement of the subject's eyes on it in the two conditions of no cue and double cue. To calculate the orienting network score, we considered the time difference between the display of the stimulus and the placement of the subject's eyes on it in the two conditions of valid spatial cue and double cue.

$\text{Modified alerting}=\left(\mathrm{eye}\;\text{fixation time}\right)\;\mathrm{no}\;\mathrm{cue}-\left(\mathrm{eye}\;\text{fixation time}\right)\;\text{double}\;\mathrm{cue}.$

$\text{Modified orienting}=\left(\mathrm{eye}\;\text{fixation time}\right)\;\text{double}\;\mathrm{cue}-\left(\mathrm{eye}\;\text{fixation time}\right)\;\text{valid spatial}\;\mathrm{cue}.$

$\text{Modified executive}=\left(\text{Modified}\;\mathrm{RT}\right)\;\text{incongruent}-\left(\text{Modified}\;\mathrm{RT}\right)\;\text{congruent}.$

Eyefixation time=the time interval starting from the appearance of the targetstimuli to the moment thesubject'seyefixatesonit.

ModifiedRT=the time interval starting from the fixation of the subject'seye on the target to their response.

The reaction time was divided into two components, eye fixation time and modified RT, based on the EOG signal. The first part highlights the prominence of the orienting and alerting networks, while the second part emphasizes the executive network.

In addition to the mentioned scores, there are other scores that can be calculated to evaluate the efficiency of the executive and orienting networks in this task. For the executive network, the ANT‐R can be used to calculate the effects of flanker conflict and location conflict. In this study, the executive score was determined based on the effect of flanker conflict.

Regarding the orienting network, the ANT‐R can be used to calculate the effects of validity, disengaging, orienting time, and moving + engaging. While the effect of validity is of utmost significance, in this study, the orienting network score was based on the moving + engaging effect. This effect has been extensively investigated in previous studies and allows for better comparability across different studies. Furthermore, in addition to the expected effects related to the orienting network, the validity effect may also include effects related to the oddball or mismatch phenomena. Therefore, the moving + engaging effect was selected as the orienting network score. Moreover, this task enables the calculation of scores related to the interaction of these effects. In the present study, in addition to the selected scores for the attention networks, the validity and location conflict scores were also calculated and reported. These scores were calculated as follows:

Validity=RTinvalidcue–RTvalidcue.

Location conflict=RTlocation incongruent–RTlocation congruent.

$\text{Modified validity}=\left(\text{eye fixation time invalid}\right)\mathrm{cue}–\left(\mathrm{eye}\;\text{fixation time}\right)\;\text{valid}\;\mathrm{cue}.$

$\text{Modified location conflict}=\left(\text{Modified}\;\mathrm{RT}\right)\;\text{location incongruent}–\left(\text{Modified}\;\mathrm{RT}\right)\;\text{location congruent}.$

Location congruent=the condition where the locationwhere the arrows appear and the direction of the centralarrow are the same.

Location incongruent =the condition where the locationwhere the arrows appear and the direction of thecentral arrow are not the same.

One of the samples was excluded from the study because it exhibited a behavioral accuracy that was more than three standard deviations below the mean accuracy of the group, as well as a reaction time that was more than three standard deviations above the mean reaction time of the group.

### Signal analysis

The EOG signal analysis was conducted using the EEGLAB toolbox (Delorme & Makeig, 2004) in MATLAB 2018b. Given the spatial distribution of the stimuli, we examined the impact of eye movement time on our assessment of cognitive functions related to attention networks. To accomplish this, we extracted the time when the subject's eyes were fixated on the presented target stimulus from the recorded EOG signal.

To prepare the data, the EOG signal was initially filtered using a band‐pass filter with a range of 1–30 Hz and re‐referenced to the right eye bottom electrode. Subsequently, the data were epoched based on the time of target stimulus appearance, ranging from −1.5 to 2 s. A baseline period was defined from −1500 to −800 ms. Finally, the moment when the subject's eyes were fixated on the stimulus was manually extracted for each subject and trial. While this time could be easily evaluated in many trials, there were some trials where it could not be determined. In trials where the subject's eyes were already fixated on the stimulus location before its presentation, the moment of eye placement was considered the time of stimulus appearance.

Based on the electrode arrangement and the conducted analysis, it was observed that during horizontal eye movements, the signals from the two channels changed in opposite directions, while during blinking or vertical eye movements, the signals from the two channels changed in the same direction. Consequently, this information enabled us to identify the timing and direction of horizontal eye movements, as well as the moment when the eyes were fixated on the target.

**FIGURE 2:**
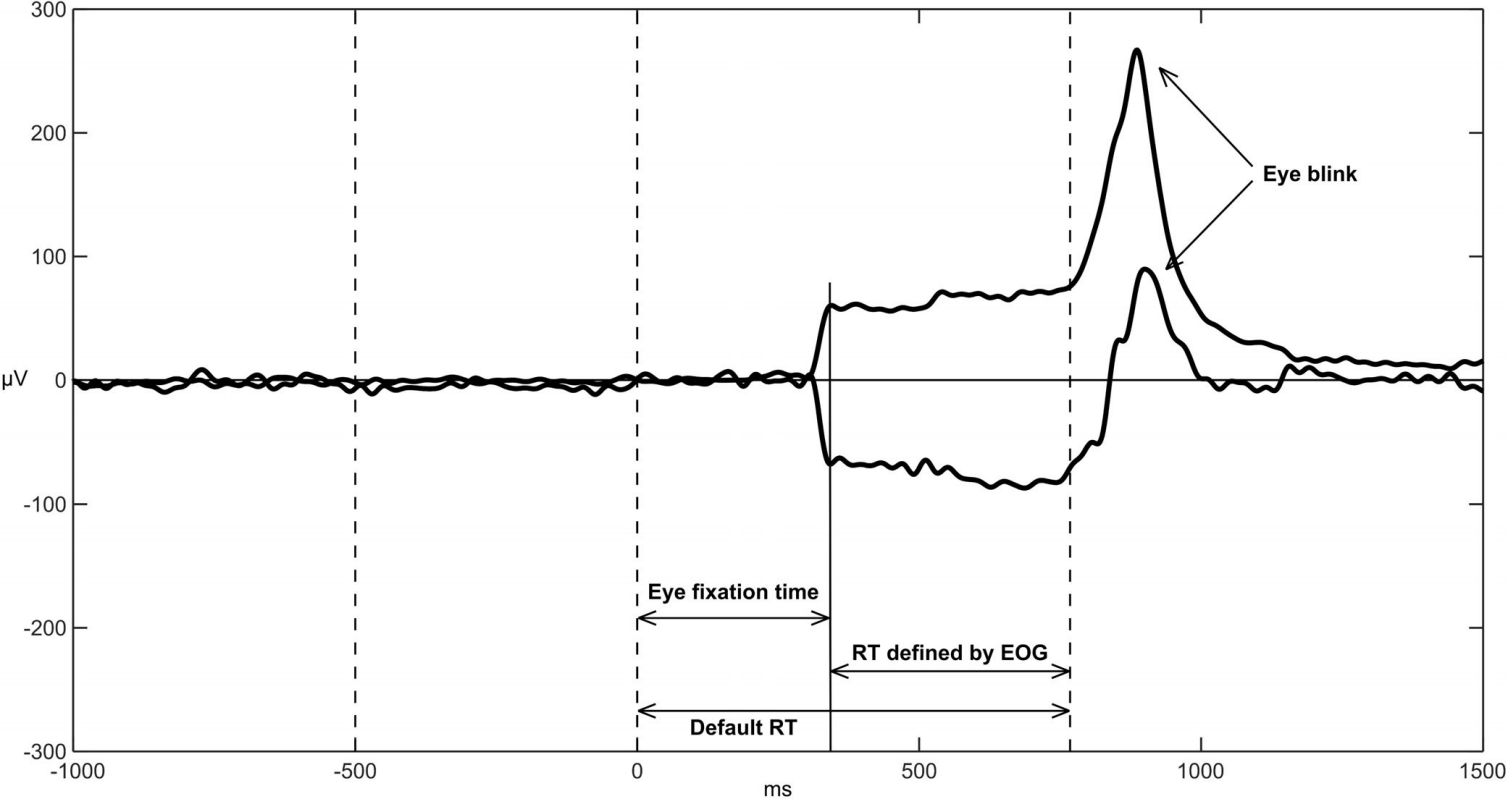
Example of an electrooculogram (EOG) trial. The displayed signals correspond to the two electrodes positioned at the outer corners of the left and right eyes. These signals were filtered and re‐referenced to the electrode at the bottom of the right eye. The baseline of the signals was established prior to the appearance of cues and subtracted from them. By arranging the electrodes strategically and conducting the analysis, horizontal eye movements result in opposite changes in the signals of the two channels, while blinking causes an increase in the signals of both channels. Consequently, the timing and pattern of horizontal eye movement and the moment the eyes fixate on the target can be identified. The three vertical dashed lines represent the onset of cues, target presentation, and the subject's response times from left to right. The figure illustrates the representation of reaction time, eye fixation time, and reaction time from eye fixation to subject response time (modified RT).

To determine the specific moment of eye placement on the target, we utilized additional information such as cue presentation time, target appearance time, and subject's response time for each trial. Furthermore, in each trial, we were aware of whether the eye movement was directed towards the target or in the opposite direction. Figure 2 provides an example illustrating a sample of trials.

### Statistical analysis

To examine the presence of a significant effect (difference from zero) in all three attention networks, we conducted analyses both with and without considering eye movement time. We estimated the effect size of the differences between the scores of each network and zero. On the single subject scale, we measured the effect size of the time differences between conditions that were used to calculate the scores of each network, both with and without considering eye movement time. We then investigated the impact of eye movement time on the effect size. Additionally, we tested the independence of attention networks from each other, with and without accounting for eye movement time, using correlation analysis.

In our statistical analysis, a *p*‐value of .05 was considered as the threshold for determining significant differences between means or significant correlations. All statistical analyses were conducted using MATLAB. The correlation coefficient, *r*, was utilized to assess effect sizes in correlation analysis, while Cohen's *d* index was employed to determine effect sizes in mean differences. For parameter *d*, effect sizes of 0.1, 0.2, 0.5, 0.8, 1.2, and 2 were classified as very small, small, medium, large, very large, and huge, respectively. Additionally, effect sizes were assessed based on the proximity to the values of 0.1, 0.3, and 0.5 for *r*, representing small, medium, and large effect sizes, respectively. Our comparison and judgment criteria were based on the degree of proximity to these effect size values.

## RESULTS

### Behavioral efficiency

The mean reaction time of the subjects, from the appearance of the target stimulus to their response, was found to be 682 ± 81 ms. Additionally, the mean reaction time from the subjects' eye placement on the stimulus to their response was 500 ± 73 ms. Moreover, the mean accuracy rate was estimated to be 95.4% ± 2.3%. It is worth noting that in 77.5% ± 11.4% of the trials, it was possible to determine the moment when the subjects fixated their eyes on the target, and their responses were correct. The mean reaction time, modified reaction time, and response accuracy in different conditions are presented in Table 1. Only trials with correct responses were included in the calculation of reaction times.

**TABLE 1:** Mean reaction times, modified reaction times, and eye fixation times of correct responses, and mean accuracy of responses to target stimuli, in different cue and congruency conditions.

	Cue type			
	None	Double	Valid spatial	Invalid spatial
Mean RT (ms) ± *SD*				
Congruent	669 ± 97	587 ± 78	623 ± 76	687 ± 83
Incongruent	802 ± 87	700 ± 91	754 ± 87	812 ± 89
Mean modified RT (ms) ± *SD*				
Congruent	361 ± 88	361 ± 67	334 ± 71	324 ± 87
Incongruent	441 ± 114	430 ± 90	415 ± 87	399 ± 98
Eye fixation time (ms) ± *SD*				
Congruent	348 ± 74	278 ± 58	216 ± 72	296 ± 57
Incongruent	347 ± 79	279 ± 95	234 ± 89	303 ± 65
Mean accuracy (%) ± *SD*				
Congruent	98.2 ± 3.1	98.3 ± 3.1	98.3 ± 2.1	97.9 ± 3.1
Incongruent	92.4 ± 7	94.7 ± 8.7	91.8 ± 4.3	91.8 ± 6.9

#### Alerting effect

The alerting effect, represented by the reduction in reaction time in the double cue condition compared to the no cue condition, was measured at Alerting = 61 ± 38 (*t*(38) = 9.98, Cohen's *d* = 1.6, *p* < .001). Additionally, the eye fixation time was reduced in the double cue condition, with a value of Modified alerting = 43 ± 32 (*t*(38) = 8.48, Cohen's *d* = 1.4, *p* < .001). Notably, there was no significant difference in the subjects' response accuracy between the no cue and double cue conditions (−0.2 ± 6.2, (*t*(38) = −0.2, Cohen's *d* = −0.03, *p* = .86).

#### Orienting effect

The response time was significantly lower in the valid spatial cue condition compared to the double cue condition, with a value of Orienting = 45 ± 30 (*t*(38) = 9.19, Cohen's *d* = 1.5, *p* < .001). Furthermore, the eye fixation time was shorter in the valid spatial cue condition, with a value of Modified orienting = 77 ± 37 (*t*(38) = 13.1, Cohen's *d* = 2.1, *p* < .001). A significant difference was observed in response accuracy between these two conditions, with a value of 1.5 ± 4 (*t*(38) = 2.32, Cohen's *d* = 0.4, *p* = .03). Additionally, the validity effect, representing the difference between the invalid spatial cue and valid spatial cue conditions, was calculated as a supplementary score for the orienting network. The values obtained were Validity = 91 ± 44 (*t*(38) = 12.9, Cohen's *d* = 2.1, *p* < .001) and Modified validity = 127 ± 54 (*t*(38) = 14.8, Cohen's *d* = 2.37, *p* < .001).

#### Conflict effect

The conflict or inhibition effect was observed as an increase in both reaction time and error rate in the incongruent condition compared to the congruent condition. The mean difference in response accuracy percentage between these two conditions was estimated at 4.8 ± 4.2 (*t*(38) = 7.27, Cohen's *d* = 1.2, *p* < .001). The mean value of the conflict effect (RT incongruent – RT congruent) was Executive = 121 ± 32 (*t*(38) = 25.8, Cohen's *d* = 3.8, *p* < .001), while the mean value of the modified conflict effect ((Modified RT) incongruent—(Modified RT) congruent) was Modified executive = 123 ± 30 (*t*(38) = 25.8, Cohen's *d* = 4.1, *p* < .001). Additionally, the Location conflict effect, calculated as a supplementary score for the executive network, showed no significant differences between the location incongruent and location congruent conditions, with values of Location conflict = 4 ± 21 (*t*(38) = 1.3, Cohen's *d* = 0.12, *p* = 0.19) and Modified location conflict = 3 ± 22 (*t*(38) = −0.7, Cohen's *d* = −0.11, *p* = .47).

### The difference in reaction times between different conditions at the single subject level

To assess the effect of different conditions on reaction times at the single subject level, we calculated Cohen's *d* index as the effect size for the differences in reaction times between cue or target conditions within each participant. For the alerting network efficiency (double cue and no cue), the mean effect size across participants was 0.43 ± 0.24. Similarly, for the modified alerting effect, the mean effect size across individuals was 0.44 ± 0.3.

Furthermore, for the orienting network efficiency (valid spatial cue and double cue), the mean effect size across participants was 0.34 ± 0.21, while the mean effect size for modified orienting was estimated at 0.67 ± 0.29.

Similarly, for the executive network efficiency (congruent and incongruent), the mean effect size across subjects was 0.87 ± 0.22, and the mean effect size for modified conflict was calculated at 1.01 ± 0.24.

To compare the effect sizes associated with each network between the two assessment methods, paired *t*‐tests were conducted. For the alerting network, there was no significant difference in the effect size between the two methods, with and without considering eye movement (Alerting and Modified alerting) (*t*(38) = 0.18, *p* = .86).

In contrast, for the orienting network, a significant difference in effect size was observed between the two methods, with and without considering eye movement (Orienting and Modified orienting) (*t*(38) = 7.76, *p* < .001). Similarly, for the executive network, a significant difference was found in the effect size between the two methods (Executive and Modified executive) (*t*(38) = 5.92, *p* < .001). Both the orienting and executive networks showed a greater effect size of the difference in reaction times when eye movement was considered compared to when it was not.

### Correlations between attention networks

Table 2 presents the correlation coefficients and significance levels between the scores of attention networks, calculated based on the default method and proposed method. A negative correlation was observed between the score of the alerting network and the score of the orienting network. However, it is worth noting that all modified attention networks appeared to be independent of each other.

**TABLE 2:** Correlation coefficients between attentional networks scores.

	Alerting	Orienting	Executive	Modified alerting	Modified orienting	Modified executive
Alerting						
Orienting	−0.50**					
Executive	−0.11	0.05				
Modified alerting	0.19	0.14	−0.02			
Modified orienting	−0.12	0.45*	−0.28	−0.08		
Modified executive	−0.20	−0.02	0.85***	−0.03	−0.24	

* Significant at *p* = .004;

** Significant at *p* = .0012;

*** Significant at *p* < .001.

## DISCUSSION

This study aimed to investigate attention networks and their interrelationships. Additionally, it examined the impact of eye movement time and its consideration on the assessment of attention network efficiency, which, to our knowledge, has not been previously explored. The behavioral results of the task revealed significant effects of conflict, alerting, and orienting in both conditions, with and without considering eye movement time (all *p* < .001). These findings align with previous studies that have reported significant differences from zero for each network (Fan et al., 2002; 2005; 2009; Luna et al., 2021; Markett et al., 2022; Wang & Guo, 2020).

The congruency of the target had a positive impact on the reduction of reaction time from the moment of target stimulus appearance and eye placement on the stimulus, as well as the error percentage, compared to the incongruent condition. Considering eye movement in calculating the conflict effect slightly increased the effect size of this effect (from *d* = 3.8 to *d* = 4.1). Moreover, the presentation of a double cue, compared to the no cue condition, decreased reaction time in both conditions, regardless of whether eye movement time was considered. When calculating the alerting effect using the time interval between the appearance of the target stimulus and eye placement on the target stimulus, the effect size was slightly reduced (from *d* = 1.6 to *d* = 1.4) compared to when the time interval between stimulus appearance and response was used. Additionally, the valid spatial cue presentation condition resulted in lower reaction times and error percentages compared to the double cue presentation condition, in both conditions with and without considering eye movement time. When calculating the orienting network score using the interval between target stimulus appearance and eye placement time, the orienting effect size increased moderately compared to when the time interval between stimulus appearance and response was used (from *d* = 1.5 to *d* = 2.1).

At the single subject level, the mean effect sizes of the alerting and modified alerting networks were found to be medium. There was no significant difference in the effect sizes of these networks for different individuals (*p* = .86). In contrast, the mean effect sizes of the orienting and modified orienting networks varied, with the former being small and the latter being large. Considering eye movement led to a significant increase in the effect sizes of this network (*p* < .001). Similarly, the mean effect sizes of the executive and modified executive networks were large and very large, respectively. Once again, considering eye movement resulted in a significant increase in the effect sizes (*p* < .001). Overall, calculating the scores of attention networks using the proposed method generally yielded greater differences between the pairs of conditions used in the calculation of each network's score.

As depicted in Table 2, there was a correlation between the alerting and orienting scores. Previous studies have shown results regarding the correlation between these networks in different populations, including normal individuals, musicians, and Parkinson's disease patients (Kalia et al., 2018; Lehtonen, 2007; Medina & Barraza, 2019; Ren et al., 2021; Vig, 2018; Wu et al., 2021). However, the results of previous studies were inconsistent with each other and with those of the present study. In this study and some others (Medina & Barraza, 2019; Ren et al., 2021; Wu et al., 2021), the alerting and orienting networks exhibited a negative correlation, whereas other studies reported a positive correlation (Kalia et al., 2018; Lehtonen, 2007; Vig, 2018). A meta‐analysis study by MacLeod et al. (2010) observed a very small, significant positive correlation between these networks, with an effect size of only 0.06, which is considered to be smaller than small.

However, according to Table 2 and the proposed method, none of these networks showed significant correlations with each other. The primary study on the ANT and its revised version demonstrated the independence of attention networks (Fan et al., 2002; 2009). Similarly, other studies have also revealed the independence of attention networks from one another (Fan et al., 2005; 2007; Gooding et al., 2006; Huang et al., 2021; Ishigami & Klein, 2010; LaCroix et al., 2021; Sørensen et al., 2019; Wang et al., 2005). The lack of significant correlations between attention networks may be attributed to the purification of each network's score.

Furthermore, Table 2 shows significant correlations between the executive and modified executive scores, as well as the orienting and modified orienting scores. The correlation between the executive and modified executive scores was higher compared to the orienting and modified orienting scores. The score of the modified alerting network did not show a significant correlation with the score of the alerting network. This finding is noteworthy because the alerting network exhibited the smallest difference in effect sizes between the two calculation methods among the attention networks. Additionally, unlike the other two networks, there was no significant difference in the effect sizes of subjects for the alerting score and modified alerting score. The absence of a significant correlation may indicate that the alerting network is evaluated differently in the two methods, with and without considering eye movement.

In the primary study of the ANT by Fan et al. (2002) and other related studies (Ishigami et al., 2016; Langner et al., 2023), the reliability of the test has been discussed. However, it is possible that better and more reliable results can be obtained by calculating the scores of attention networks using different approaches, such as using a trimmed reaction time (McConnell & Shore, 2011) or using dispersion parameters of reaction time or the method proposed in this work.

One limitation of this study was the inability to precisely determine the moment of eye placement on the stimuli for all trials and the uncertainty regarding the sequence of processes occurring throughout the trial. This can be addressed in future studies by utilizing eye‐tracking technology. Another limitation was that despite dividing the reaction time to isolate the effects of the networks, there are still other factors present within each time interval that may not be directly related to the attention networks. For instance, the time interval between selecting the direction of the central arrow and making a keypress decision, as well as the time interval between making the keypress decision and the actual keypress, may not be directly related to the attention networks. Nevertheless, these time intervals were taken into account when calculating the scores for attention networks. In order to eliminate the influence of these time intervals and obtain more accurate and pure scores for the attention networks, electroencephalography (EEG) could potentially be employed. Overall, by addressing these limitations and exploring alternative calculation methods, further advancements can be made to enhance the reliability and accuracy of assessing attention networks.

## CONCLUSION

This study highlights the importance of considering eye movement time when calculating the efficiency of attention networks. The findings suggest that this consideration can lead to different evaluations of these networks, potentially explaining the inconsistencies observed in previous studies. Therefore, further research in this area is warranted.

Furthermore, the method presented in this study supports the notion of independence among attention networks, which aligns with the findings of earlier studies, including the original ANT study. In contrast, the scores of attention networks were found to be interdependent using the standard method. Consequently, purifying the evaluation of attention network efficiency can offer a clearer understanding of their nature. Future studies can benefit from more accurate evaluations of eye movement time by utilizing eye tracking technology. This approach can contribute to a more refined purification of attention network scores. Additionally, exploring the possibility of further purifying the evaluation of attention network efficiency by dividing the time intervals of modified reaction time or eye fixation time into subintervals may provide deeper insights into these networks.

## CONFLICT OF INTEREST STATEMENT

The authors declare no conflicts of interest.

## ETHICS STATEMENT

Informed consent forms were obtained from all participants. The present study received approval from the Ethics Committee of Mashhad University of Medical Sciences.
